# Comparison of Communication Channels for Large-Scale Type 2 Diabetes Risk Screening and Intervention Recruitment: Empirical Study

**DOI:** 10.2196/21356

**Published:** 2021-09-09

**Authors:** Kari Jalkanen, Riia Järvenpää, Tanja Tilles-Tirkkonen, Janne Martikainen, Emma Aarnio, Reija Männikkö, Eeva Rantala, Leila Karhunen, Marjukka Kolehmainen, Marja Harjumaa, Kaisa Poutanen, Miikka Ermes, Pilvikki Absetz, Ursula Schwab, Timo Lakka, Jussi Pihlajamäki, Jaana Lindström, The StopDia Study Group

**Affiliations:** 1 School of Pharmacy, Faculty of Health Sciences University of Eastern Finland Kuopio Finland; 2 Department of Public Health Solutions Finnish Institute for Health and Welfare Helsinki Finland; 3 School of Medicine, Institute of Public Health and Clinical Nutrition University of Eastern Finland Kuopio Finland; 4 Department of Medicine, Endocrinology and Clinical Nutrition Kuopio University Hospital Kuopio Finland; 5 VTT Technical Research Centre of Finland Ltd Espoo Finland; 6 Clinical Nutrition and Obesity Center Kuopio University Hospital Kuopio Finland; 7 See Acknowledgments

**Keywords:** communication, digital tool, prevention, public health campaign, risk identification, screening, social media, study recruitment, type 2 diabetes, mobile phone

## Abstract

**Background:**

Clinical trials have shown that type 2 diabetes (T2D) is preventable through lifestyle interventions targeting high-risk people. Nevertheless, large-scale implementation of risk identification followed by preventive interventions has proven to be challenging. Specifically, recruitment of participants into preventive interventions is an important but often overlooked part of the intervention.

**Objective:**

This study aims to compare the reach and yield of different communication channels to engage people at increased risk of T2D to fill in a digital screening questionnaire, with emphasis on reaching those at most risk. The participants expressing their willingness to participate is the final step in the risk screening test, and we aim to determine which channels had the most participants reach this step.

**Methods:**

We established a stepwise web-based T2D risk screening tool with automated feedback according to the T2D risk level and, for those who were eligible, an invitation to participate in the StopDia prevention intervention study conducted in a primary health care setting. The risk estimate was based on the Finnish Diabetes Risk Score; history of repeatedly measured high blood glucose concentration; or, among women, previous gestational diabetes. We used several channels to invite people to the StopDia web-based screening tool, and respondents were classified into 11 categories based on the channel through which they reported having learned about StopDia. The demographics of respondents reached via different communication channels were compared using variance analysis. Logistic regression was used to study the respondents’ likelihood of progressing through risk screening steps.

**Results:**

A total of 33,399 persons started filling the StopDia screening tool. Of these, 86.13% (28,768/33,399) completed the test and named at least one communication channel as the source of information about StopDia. Altogether, 26,167 persons filled in sufficient information to obtain risk estimates. Of them, 53.22% (13,925/26,167) were at increased risk, 30.06% (7866/26,167) were men, and 39.77% (10,136/25,485) had low or middle education levels. Most frequently mentioned channels were workplace (n=6817), social media or the internet (n=6712), and newspapers (n=4784). The proportion of individuals at increased risk was highest among those reached via community pharmacies (415/608, 68.3%) and health care (1631/2535, 64.33%). The communication channel reaching the largest percentage of interested and eligible men (1353/3979, 34%) was relatives or friends. Health care (578/1069, 54.07%) and radio or television (225/487, 46.2%) accounted for the largest proportion of people with lower education.

**Conclusions:**

Communication channels reaching a large number of people, such as social media and newspapers, were the most effective channels for identifying at-risk people. Personalized approaches increased the engagement of men and less-educated people. Community pharmacies and health care services reached people with a particularly high T2D risk. Thus, communication and recruitment channels should be selected and modified based on the intended target group.

**International Registered Report Identifier (IRRID):**

RR2-10.1186/s12889-019-6574-y

## Introduction

### Background

Diabetes is one of the most common noncommunicable diseases and affects 10%-15% of adult populations in different countries, and most patients have type 2 diabetes (T2D) [1]. Furthermore, T2D can remain undiagnosed for several years, and a considerable proportion of people with T2D are not aware of their disease [2,3]. Poorly controlled or untreated T2D can lead to serious micro- and macrovascular complications [4,5], and the treatment of these comorbidities accounts for most of the costs related to T2D [6].

Evidence from studies conducted among different populations has shown that T2D is preventable by providing lifestyle interventions to people at increased risk [7-10]. To identify people who are at risk and would thus benefit from lifestyle interventions, several risk scores have been developed [11]. One of the most widely used tools is the Finnish Diabetes Risk Score (FINDRISC) [10]. It includes 8 questions, 4 of which deal with modifiable risk factors (BMI; waist circumference; consumption of vegetables, fruits, and berries; and physical activity). Thus, in addition to being a risk screening tool, the FINDRISC can also be considered as a brief intervention to increase awareness of T2D prevention possibilities [12].

Despite the research evidence of the efficacy of lifestyle interventions, large-scale implementation of risk identification followed by preventive interventions has proven to be challenging. A common shortcoming is that participant enrollment is often seen as a preliminary phase that precedes the actual intervention. In reality, successful recruitment may determine the outcome and effectiveness of the entire intervention. In interventions including screening and participant recruitment, the PIPE (Penetration, Implementation, Participation, and Effectiveness) framework for designing and evaluating health promotion programs provides steps that can be identified [13]. First, as many people as possible need to be made aware of and interested in taking up the screening (reach). Second, the respondents who are at risk need to be motivated to participate in the intervention (yield). Furthermore, preventive interventions do not always reach the right target group. For example, men and people with lower socioeconomic status are known to be more susceptible to diabetes, yet they tend to be less represented in prevention programs [14-17]. Nevertheless, there is evidence to suggest that people with lower socioeconomic status can benefit equally from lifestyle interventions, if only they can be reached and enrolled to participate [17,18].

Few studies have been published that compared different communication strategies to identify individuals at increased risk for T2D [19-21]. New methods such as mobile technology and social media are currently complementing traditional methods in the recruitment of study participants to intervention studies [22-26]. The COVID-19 pandemic has created an unprecedented need for web-based solutions, including the recruitment of research participants [27,28]. Previous studies have, to a large extent, analyzed traditional recruitment methods or few web-based solutions.

Stop Diabetes (StopDia) was a large-scale, multidisciplinary study on the prevention of T2D [29] conducted during 2016-2019 in Finland. One of the main aims of StopDia was to increase the coverage of screening and recruitment of people at increased risk for T2D. Using a web-based screening and recruitment tool allowed us to analyze the differences in the effects of communication channels in a substantially larger participant pool than in previous studies.

### Objectives

In this study, we aim to compare the reach and yield of different communication channels in engaging people to fill in a digital screening questionnaire and to express their interest in taking part in the StopDia randomized controlled trial (RCT). Furthermore, we explore the potential of different channels to reach the underrepresented population groups and demographic groups that previous research has indicated as being at the highest risk of T2D, such as men [4] and people with lower education [30].

## Methods

### Context

This study is a part of the StopDia RCT (*NCT03156478*) to investigate T2D prevention with lifestyle counseling delivered via a mobile app alone or in combination with a group setting [29]. The study is based on anonymized data collected during the study participant recruitment phase of the RCT from users of the StopDia web-based risk screening tool.

The methodology of the project as a whole and regarding the development of the recruitment strategy was based on the Self-Determination Theory (SDT) [31]. The SDT comprises a continuum from external factors of motivation to internal factors, such as enjoyment, personal values, perception of autonomy and self-efficacy, and relatedness.

The recruitment campaign brand and tone of voice was aimed at creating positive, relatable feelings, particularly for our target audience. Evidence-based tactics such as using an informal tone of voice, avoiding medical and moralizing terminology, including visual content, and creating an easy-to-use design at the screening tool were used [32,33].

The key messages on the recruitment campaign were tailored and targeted for the primary audience, known to be at elevated risk, and also hard to reach to health interventions: men with middle or lower levels of education. We tested the contents in social media and optimized the contents and communication channels accordingly.

The interactive and stepwise web-based risk screening tool was available in Finnish on the StopDia website [34]. The risk screening tool could be filled in by anyone entering the site, and the tool provided the users with automated feedback on their risk level. The participants for the StopDia RCT were recruited from the provinces of North Savo, South Karelia, and Päijät-Häme in Finland during the 12-month period from March 1, 2017, to February 28, 2018. Thus, only persons who entered a postal code matching the study region were eligible and included in this study.

Respondents’ answers to the questions of the web-based risk screening tool, as well as the date and time of screening completion, were saved to a database. We were not able to collect respondents’ contact or identification information at this stage because of the obligatory face-to-face informed consent to participate and agreement to data collection in the clinical trial. Several responses from the same IP address were allowed, acknowledging the fact that the same device could be used by several people, for example, in public service facilities. The IP addresses were saved to a database but were not used in the study. The site visitors were informed that filling in the risk screening questionnaire on the website was considered as consent to use the anonymized data in the research. The Research Ethics Committee of the North Savo Hospital District has processed the ethical application and granted a permit (467/2019) to perform the study.

### StopDia Digital Screening Tool

**Figure 1:**
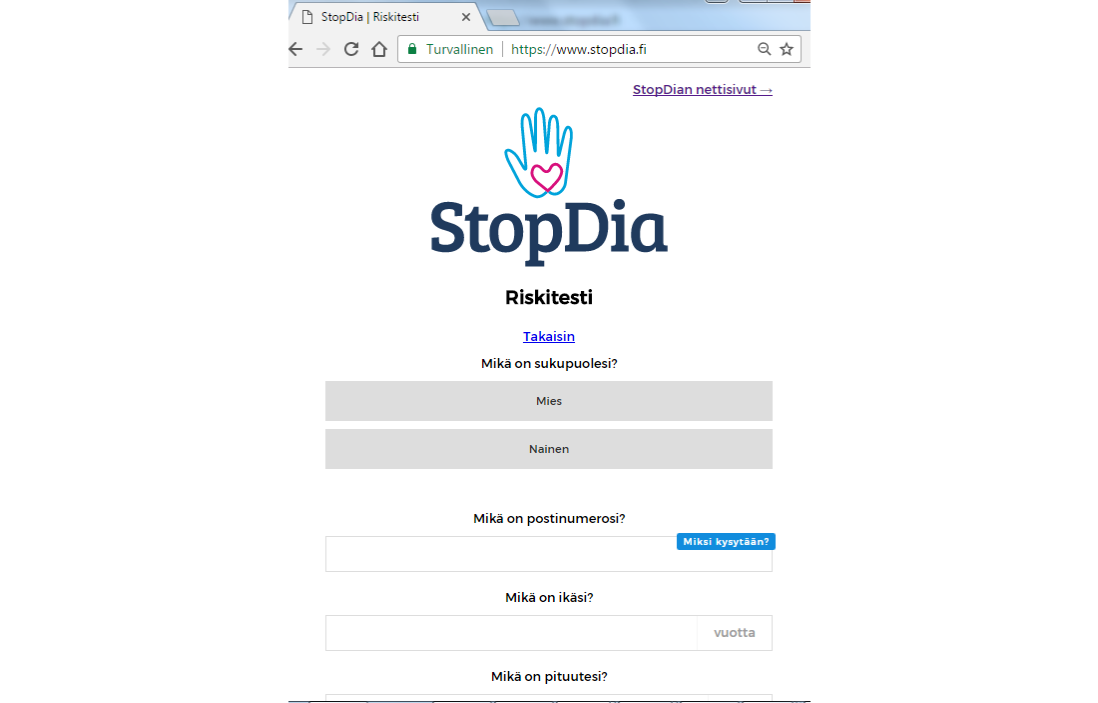
The StopDia web-based type 2 diabetes risk screening tool, starting with questions on sex, postal code, and age.

The web-based risk screening tool was available on the website [34] and could be accessed by a web browser on a desktop computer or a mobile device. The tool contained the FINDRISC T2D risk questionnaire and some additional questions (Figure 1). The FINDRISC is a validated, self-administered questionnaire used to calculate a score that gives an estimate of the respondents’ 10-year risk of developing T2D [10]. It is composed of questions on age (in years); BMI (calculated by dividing weight [kg] with height [m] squared); waist circumference (cm); the intake of fruits, berries, and vegetables (daily or not daily); physical activity (at least 30 minutes per day or less than 30 minutes per day); blood pressure medication (yes or no); history of high blood glucose concentration (yes or no); and family history of diabetes (no family history; grandparent, aunt, uncle or first cousin, but no own parent, brother, sister or child; or parent, brother, sister or own child). A total FINDRISC score of at least 12 of the maximum 26 was defined to indicate an increased risk of T2D. The additional questions were about sex (male or female), previous gestational diabetes in women (yes or no), high blood glucose (fasting glucose concentration of 6.1-6.9 mmol/L or 2-hour glucose concentration of 7.8-11.0 mmol/L in the oral glucose tolerance test) measured repeatedly in the past (yes or no), educational level (university, college, vocational school, high school, or elementary school), and respondents’ own perception of their risk of T2D (very low, low, average, high, or very high). In the analysis, universities and colleges were classified as providing high education.

The StopDia RCT inclusion criteria, in addition to living in the study area and having an increased risk for T2D, were age (eligible if 18-70 years); the possibility of using computers, smartphones, or tablets with internet connection (yes or no); and having own email address (yes or no). Exclusion criteria were prevalent diabetes (no, type 1 diabetes, T2D, or diabetes of unknown type), pregnancy (yes or no), and cancer treatment within the past 6 months (yes or no). These criteria defined the respondents’ progress through the steps of the risk screening tool. Respondents who were excluded but were at increased T2D risk based on their answers received a web-based information brochure and instructions to contact their health care services for guidance. The respondents did not receive financial or other compensation to fill in the form.

Furthermore, the communication channel through which respondents had learned about StopDia was enquired (“Where did you learn about StopDia?”). The respondents could state their communication channel by selecting one or many of the 11 predetermined categories or they could provide a free-text answer. The participants were also asked, “Did someone specifically ask you to fill in the StopDia digital screening tool?” with predefined options (health care professional, pharmacist, relative, colleague or boss, or nobody). A free-text answer was also an option for this question.

Finally, participants who were deemed eligible to participate were asked whether they would *be interested* in participating in the StopDia study. Those who replied “yes” were shown the StopDia three-page study information letter and consent forms, after which they were asked whether they *were willing* to participate in StopDia. As it became apparent early during the recruitment, one-third of respondents left the site at this point; to increase engagement, we decided to change the final step slightly by replacing the question “Are you willing to participate in StopDia lifestyle intervention study?” with a less decisive question, “Would you like to get the instructions to make an appointment with the StopDia study nurse?” As stated earlier, we were not able to collect information on who actually booked an appointment with the study nurse.

### Strategies to Reach Possible Participants

We collaborated with local public organizations to disseminate information about the StopDia study and to enhance risk identification at nurse and physician appointments, dental care, maternity services, occupational health care, and social services. Collaboration was established with pharmacies in the study areas, and 31 pharmacies arranged T2D screening days. Other collaborators included patient associations, nongovernmental organizations (NGOs), and employers. The study group regularly posted content on social media (Facebook, YouTube, Twitter, and Instagram) and paid for social media visibility (both promoted posts and advertisements). A summary of the campaign statistics is provided in Multimedia Appendix 1.

We sent several press releases and collaborated with local media. Up to 500 lay articles were published about the study in local and national media. To target men, we organized and participated in many local events (ice hockey games, camping, and hunting fairs). We also collaborated with local food banks to get in contact with hard-to-reach population groups with economic difficulties.

**Figure 2:**
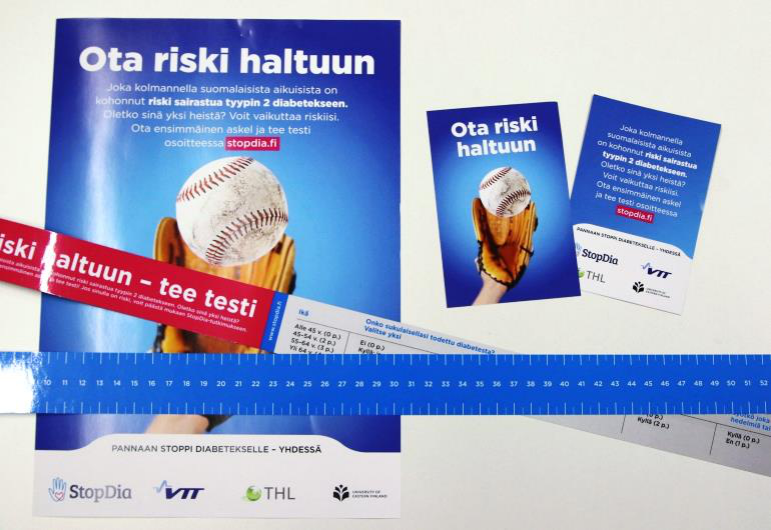
StopDia printed campaign materials: poster, flyer, measuring tape with the type 2 diabetes screening questionnaire. More than 150,000 pieces of print materials were delivered to health care and other public services, nongovernmental organizations, pharmacies, local workplaces, and shops.

**Figure 3:**
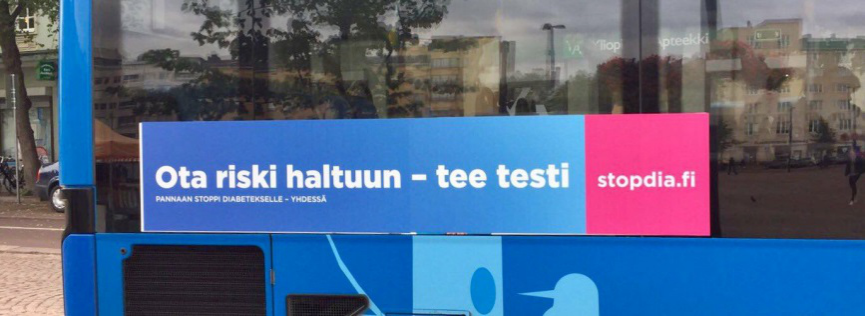
StopDia advertisement on local buses with a short version of the campaign slogan: “Take control of your risk – take the test.”

The main recruitment campaign message, “Take control of your risk – One-third of Finns are at risk of diabetes, are you?” was distributed via different communication channels. The slogan was followed by a brief explanation of the study and the screening tool web address. The aim was to use a message that emphasized self-efficacy in risk reduction. The same message along with instructions on how to participate in the study was used in print materials (a total of more than 150,000 posters, leaflets, printed FINDRISC questionnaires, and StopDia measuring tapes for measuring waist circumference with the FINDRISC questionnaire printed on it; Figure 2), advertisements on local buses (Figure 3), and in digital materials (video advertisements on collaborators’ information screens and intranet, content on social media [Facebook, Twitter, and YouTube], and targeted emails for the workforce at the partnering organizations).

The printed campaign materials were delivered to local establishments (eg, health care and other public services, NGOs, pharmacies, workplaces, and shops) and could be ordered on the StopDia website.

**Figure 4:**
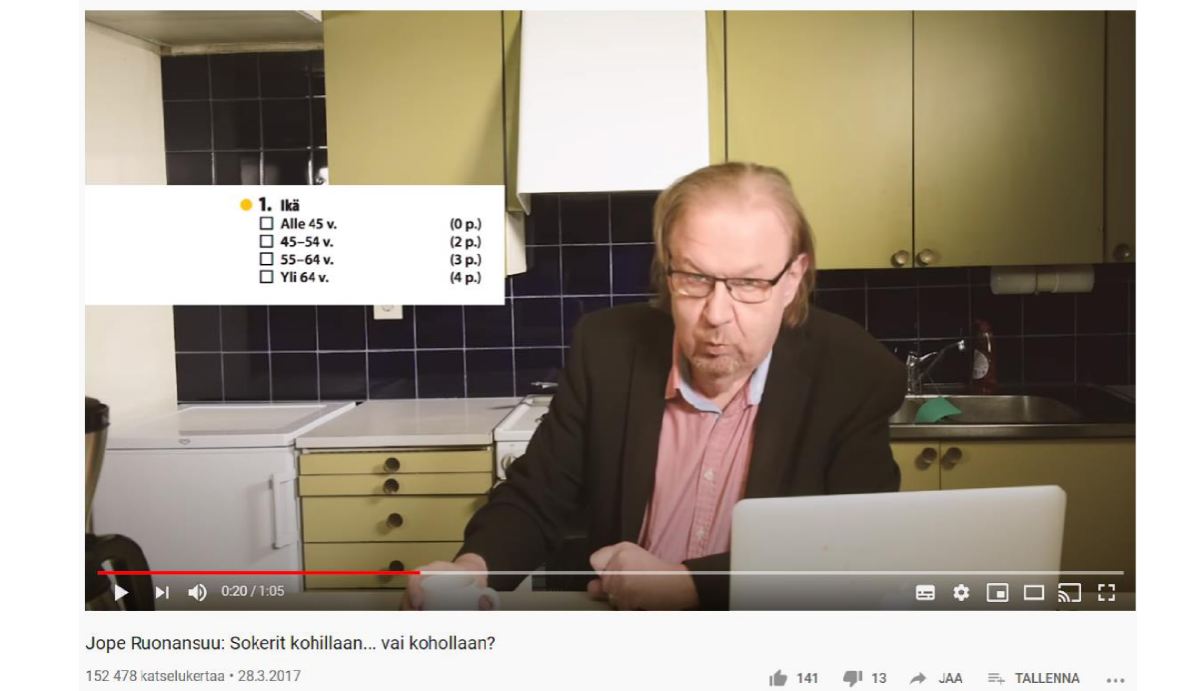
Screen capture of a StopDia marketing video with a Finnish comedian, published and promoted on YouTube, Facebook, Instagram, and Twitter. In the video, the comedian is filling in the web-based risk screening tool.

One of the key marketing materials we produced was a short video (Figure 4), in which a well-known Finnish comedian filled in the web-based risk screening tool.

**Figure 5:**
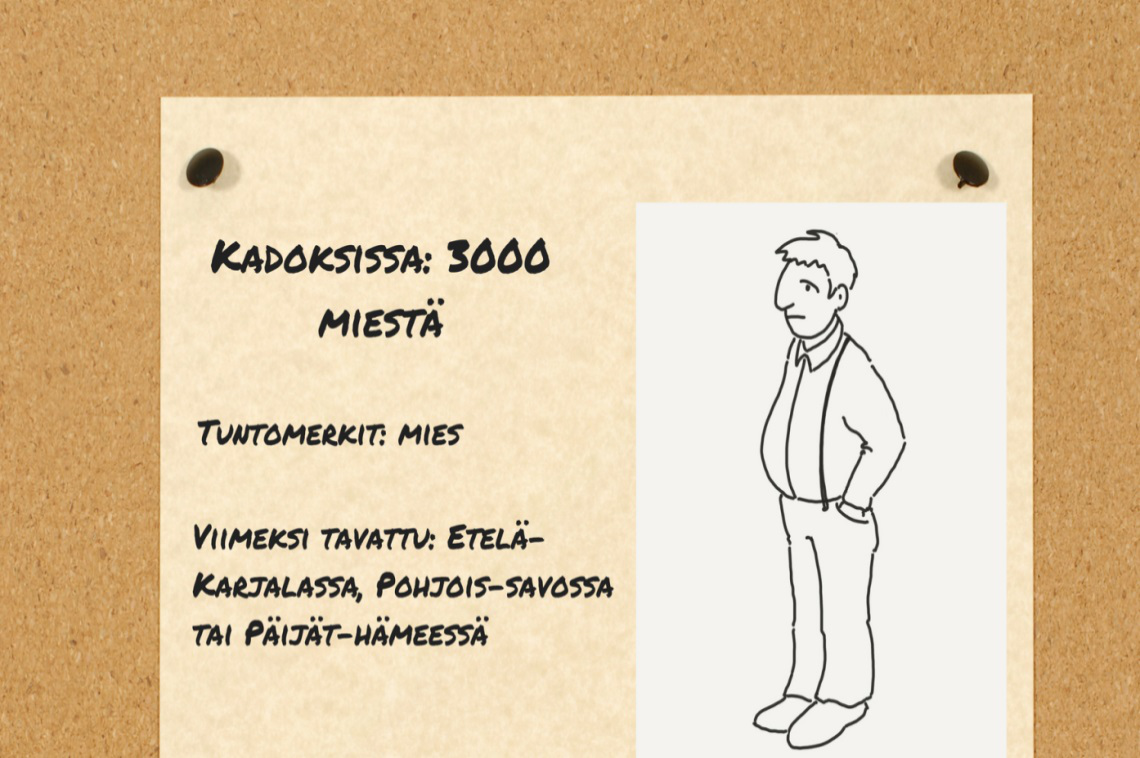
A Facebook advertisement published on local Facebook groups with the text: “Have you seen this man.” The caption of the picture says: “Lost: 3000 men. Identifying characteristics: Man. Last seen in: South Karelia, North Savo, or Päijät-Häme.” The advertisement was accompanied with the text: “If seen, please ask him to fill in the StopDia web-based risk screening tool.”.

An example of our social media campaigns is a Facebook advertisement, “Have you seen this man?” (Figure 5), aiming to reach men at T2D risk. The advertisement was promoted as a paid advertisement on Facebook and Instagram and shared by local Facebook groups. In addition, the advertisement was distributed via the StopDia stakeholder newsletter.

### Classification of Different Communication Channels

The communication channels were categorized primarily according to the respondents’ answers to the question, “Where did you learn about StopDia?” (Table 1).

Furthermore, based on the answer to the question, “Did someone specifically ask you to fill in the StopDia risk screening tool?” we categorized the recruitment process as either “active” (if the respondent mentioned someone) or “passive” (if the answer was “nobody”). These terms were adopted from previous research [35] and modified to fit this study. Specifically, we made the distinction that persons not directly associated with the research staff such as physicians, pharmacists, and relatives were considered to be active recruiters if they directly had recommended someone to participate.

**Table 1:** Categorization of communication channels based on the self-reported source of information.

Communication channel category	Predetermined answer options included in the channel category	Free-text mentions included in the channel category
Newspaper	Newspaper or magazine (web-based or print)^a^	Press and web-based newspapers
Radio or television	Radio or television^a^	Radio or television; specific television program
Workplace	My workplace^a^ Manager or colleague^b^	Workplaces, manager, coworker, work emails, schools, and universities
Pharmacy	Pharmacy^a^ Pharmacist^b^	Community pharmacy, the pharmacist
Health care	Health care professional at an appointment^a^ Health care service desk or other service desk^a^ Health care worker^b^	Physician, dentist, nurse, dietician, optician, school health care, health care center, maternity clinic, and other municipal service desks
Event	Sports event, fair, or other public event^a^	Seminar, exhibition, public event, training session, sports event, and presentation
NGO^c^	Patient organization or other organization (such as Diabetes Association or Heart Association)^a^	Patient organization, the Rotaries, labor union, and other NGOs
StopDia	StopDia study website^a^	The StopDia project itself, its webpage, and personnel, persons doing face-to-face recruitment
Social media and internet	Facebook or Twitter^a^	Twitter, Facebook, WhatsApp, YouTube, Snapchat, Tumblr, Reddit, Instagram, blogs or search engines, and named media persons
Relative or friend	Relative, friend or acquaintancea,^b^	Friend, wife, husband, daughter, son, and other family members
Other	Somewhere else, where?^a,b^ Free-text answer	Fitness advisors, swimming halls, libraries, marketplaces, buses, personal email, SMS text message, Donald Duck, and other real and imaginary characters

^a^Answer options to the question: “Where did you learn about the StopDia study (you may select multiple options)?”

^b^Answer options to the question: “Did someone specifically ask you to fill in the StopDia digital screening tool?”

^c^NGO: nongovernmental organization.

### Statistical Analyses

Answers from the respondents were checked and implausible values for body weight (<30 kg or >200 kg; n=100), waist (<59 cm or >151 cm; n=381), and height (<139 cm or >202 cm; n=81) were coded as missing values, but the other answers of these respondents were left intact and used to calculate their FINDRISC scores. These limits were chosen based on the lowest and highest measured values in a random population-based survey in Finland [36].

The demographic characteristics of the respondents who were reached via different communication channels were compared using a variance analysis. Logistic regression analysis was used to study the respondents’ likelihood of progressing through the risk screening steps (Figure 1), with “workplaces” as the reference channel, using the general linear function in RStudio with the binomial family.

Data were analyzed using SPSS Statistics version 25 (IBM Corp) and RStudio version 3.3.4 [37].

## Results

### Respondents’ Progress Through the Risk Screening Tool

**Figure 6:**
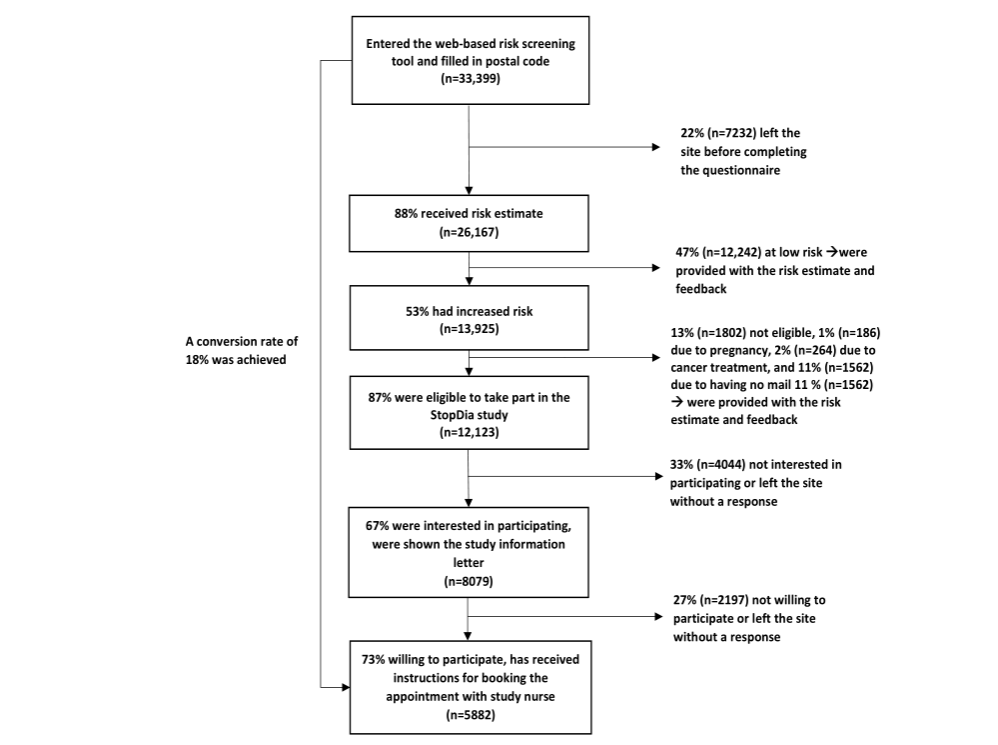
Flow diagram depicting the respondents’ progression through the stepwise StopDia web-based risk screening tool. Conversion rate is the proportion of persons who were willing to participate (n=5882) of those who entered the web-based risk screening tool (n=33,399).

The flow chart of the stepwise recruitment is presented in Figure 6.

In total, 33,399 persons with a postal code matching the study area started filling in the web-based risk screening tool.

Of these, 23.45% (7832/33,399) left the site without entering enough information to obtain a T2D risk estimate. The most frequently omitted question was waist circumference (n=3932).

Of the respondents who completed the questionnaire, 53.22% (13,925/26,167) had an increased T2D risk. On the basis of the eligibility criteria of the StopDia RCT, 12.94% (1802/13,925) respondents with increased risk were excluded, and the most common reason for exclusion was not having an email address (1562/13,925, 11.21%). Of these excluded respondents without email, 37.26% (582/1562) were men and 52.37% (818/1562) had low education, and their mean FINDRISC score was 15.3 (SD 3.2).

Altogether, 12,123 respondents were deemed eligible to participate and were asked whether they would *be interested* in taking part in the StopDia study. A total of 66.64% (8079/12,123) of the eligible, responded as being interested, and of them 72.8% (5882/8079) expressed their willingness and asked for instructions to participate in StopDia. The conversion rate of our recruitment process from reach to willingness to participate was 17.61% (5882/33,399).

### Comparison of the Reach and Yield of Different Communication Channels

A total of 28,756 respondents named at least one communication channel through which they had learned about StopDia. Of these, 8.86% (2546/28,756) named two communication channels and 1.12% (323/28,756) named three or more communication channels.

**Figure 7:**
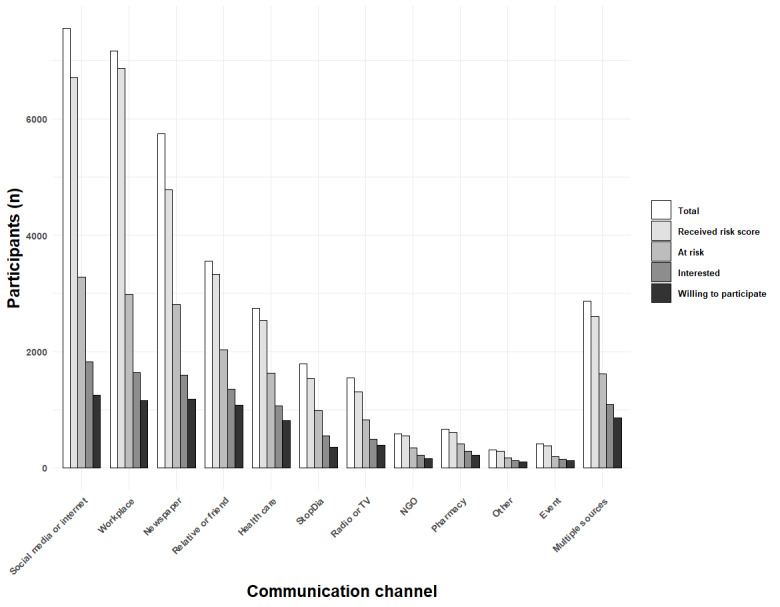
The number of participants progressing through the different steps of the risk screening tool. NGO: nongovernmental organization; TV: television.

The largest number of respondents were engaged via social media, workplaces, and newspapers (Figure 7), and the least frequently mentioned channels were events, NGOs, and pharmacies. Consequently, the highest absolute number of people at risk and interested in participating in the StopDia study were reached via social media, workplaces, and newspapers. Many individuals at increased risk were also reached through relatives and friends and via multiple communication channels.

The effectiveness of different communication channels to get people to progress through the StopDia web-based risk screening tool, receive a risk estimate, and eventually become interested in participating is presented as odds ratios (ORs), with workplace as the reference channel, as shown in Table 2. People who reached through workplaces were most likely to complete the risk screening and get an estimate of their risk, followed by NGOs and a relative or a friend. The least effective channels in this regard were newspapers and radio or television (TV). Multiple communication channels as well as health care services and pharmacies were less effective in prompting the respondents to fill in enough information to receive the risk estimate compared with the reference channel.

**Table 2:** Communication channel and likelihood (odds ratio) of progressing through the StopDia risk screening tool.

Communication channel	Filled in the questionnaire and received risk estimate, OR^a^ (95% CI)	At risk, OR (95% CI)	Eligible (if at risk), OR (95% CI)	Interested to participate (if eligible), OR (95% CI)	Agreed to participate (if interested), OR (95% CI)
Workplace	Reference	Reference	Reference	Reference	Reference
Event	0.44 (0.28-0.72)	1.31 (1.00-1.72)	1.18 (0.90-1.53)	12.04 (4.49-49.2)	1.14 (0.72-1.78)
Social media and internet	0.34 (0.29-0.39)	1.28 (1.19-1.38)	1.05 (0.97-1.13)	1.68 (1.45-1.96)	1.34 (1.15-1.56)
Newspaper	0.21 (0.18-0.24)	1.83 (1.69-1.98)	1.21 (1.12-1.31)	2.06 (1.74-2.43)	0.67 (0.57-0.80)
Pharmacy	0.50 (0.35-0.73)	2.86 (2.31-3.53)	2.11 (1.74-2.56)	3.67 (2.43-5.81)	1.74 (1.29-2.35)
Radio or television	0.22 (0.18-0.27)	2.30 (2.00-2.64)	1.42 (1.25-1.61)	2.13 (1.63-2.82)	2.25 (1.78-2.84)
Relative or friend	0.73 (0.59-0.91)	2.08 (1.89-2.30)	1.84 (1.67-2.03)	2.98 (2.43-3.68)	1.92 (1.62-2.29)
Health care	0.54 (0.43-0.67)	2.41 (2.15-2.70)	1.81 (1.62-2.01)	3.00 (2.38-3.79)	1.84 (1.52-2.22)
StopDia	0.25 (0.20-0.30)	2.32 (2.04-2.65)	1.38 (1.22-1.56)	1.29 (1.02-1.62)	1.56 (1.23-1.97)
NGO^b^	0.88 (0.52-1.64)	2.59 (2.00-3.36)	2.28 (1.79-2.91)	2.94 (1.82-5.03)	1.44 (0.99-2.10)
Other	0.50 (0.39-0.65)	1.32 (1.16-1.51)	1.17 (1.03-1.33)	1.60 (1.24-2.09)	2.24 (1.74-2.89)
Multiple channels^c^	0.39 (0.33-0.47)	2.31 (2.09-2.54)	1.80 (1.64-1.97)	3.00 (2.46-3.67)	1.74 (1.47-2.06)

^a^OR: odds ratio.

^b^NGO: nongovernmental organization.

^c^Respondents could select or mention multiple communication channels.

Of the respondents who were provided with a risk estimate, the highest likelihood of being at increased risk was among those who were reached via pharmacies, NGOs, and health care (Table 2). Respondents who mentioned the StopDia website or personnel as communication channels were also more likely to be at increased risk. The lowest likelihood of being at an increased risk was among the respondents who were reached via workplaces. The lowest likelihood of being eligible among respondents at increased risk was among those who were reached via workplaces, events, or social media.

Eligible persons whose communication channel was the workplace were least likely to express interest in participating in the StopDia study. The most efficient communication channels in this step were events, pharmacies, health care services, a relative or friend, NGOs, and multiple communication channels. Finally, of the eligible and interested, those who mentioned radio or TV as their communication channel were most likely to and those who mentioned newspapers were least likely to be willing to participate in StopDia when offered the possibility to make an appointment with the study nurse.

### Characteristics of the Respondents Reached Via Different Communication Channels

Among the 26,167 respondents who received their risk estimates, 13,925 (53.21%) were at increased risk (Table 3). The highest proportion of people at an increased risk was among those who were reached via pharmacies (415/608, 68.3%), health care (1631/2535, 64.34%), and a relative or friend (836/1306, 64.01%). Overall, 23.58% (7877/33,399) of those who received their risk estimate and 22.47% (1815/8076) of those who were interested in participating in the StopDia study were men. The proportion of men was highest among those who were reached through a relative or friend. The workplace was the least effective channel for reaching men.

**Table 3:** Characteristics of the respondents who received an estimate on their risk, by communication channel (n=26,167).

Communication channel	Total (n=26,167), n (%)	Men, n (%; 95% CI^a^)	Age (years), mean (SD)	Low or middle education, n (%; 95% CI^a^)	At risk, n (%; 95% CI^a^)
Workplace	6871 (26.25)	1136 (16.53; 15.67-17.43)	47 (11)	1776 (25.83; 24.81-26.88)	2983 (43.41; 42.25-44.59)
Social media and internet	6712 (25.65)	2269 (33.81; 32.68-34.95)	45 (14)	2896 (44.59; 43.38-45.80)	3439 (48.76; 47.57-49.96)
Newspaper	4784 (18.28)	1616 (33.78; 32.45-35.13)	51 (13)	1931 (41.46; 40.00-42.89)	2808 (58.7; 57.29-60.08)
Relative or friend	3327 (12.71)	1428 (42.92; 41.25-44.61)	50 (14)	1504 (46.12; 44.42-47.84)	2026 (60.9; 59.23-62.54)
Health care	2535 (9.68)	2535 (28.88; 27.14-30.67)	50 (15)	1213 (48.70; 46.74-50.66)	1631 (64.34; 62.45-66.18)
StopDia	1537 (5.87)	516 (33.57; 31.25-35.97)	54 (13)	647 (44.04; 41.52-46.59)	986 (64.15; 61.72-66.51)
Radio or television	1306 (4.99)	427 (32.7; 30.21-35.29)	55 (13)	600 (46.66; 43.94-49.39)	830 (63.55; 60.91-66.12)
Pharmacy	608 (2.32)	154 (25.3; 22.0-28.9)	51 (14)	236 (39.3; 35.4-43.2)	415 (68.3; 64.5-71.8)
NGO^b^	547 (2.09)	179 (31.1; 27.5-35.1)	51 (16)	200 (36.9; 32.9-41.0)	338 (61.79; 57.65-65.77)
Event	375 (1.43)	128 (34.1; 29.5-39.1)	50 (15)	136 (36.9; 32.1-41.9)	199 (53.1; 48.0-58.1)
Other	283 (1.08)	65 (23; 18.5-28.2)	51 (13)	78 (30; 24.8-35.8)	167 (59; 53-65)
Multiple channels^c^	2602 (9.94)	789 (30.32; 28.59-32.12)	50 (13)	1045 (40.87; 38.98-42.79)	1620 (62.26; 60.38-64.10)
All	26,167 (100.00)	7866 (30; 29.51-30.62)	49 (14)	10,136 (39.77; 39.17-40.37)	13,925 (53.22; 52.61-53.82)

^a^Binomial variable CIs were calculated using the Wilson method.

^b^NGO: nongovernmental organization.

^c^Respondents could select multiple communication channels and were included in all the mentioned categories.

The mean age of all respondents and of those who were interested in participating was 49 (SD 14) years and 53 (SD 11) years, respectively (Table 4). The respondents who were reached via radio or TV were the oldest and those who were reached via social media were the youngest. The overall proportion of respondents with low or middle education was 40.8% (11,275/27,632), and among those who were interested in participating in StopDia, the proportion was 37.17% (3003/8079). The largest proportion of people with low or middle education was reached through health care and a relative or friend, and the lowest proportion, through workplaces.

**Table 4:** Characteristics of the respondents who were eligible and interested to participate in StopDia, by communication channel (n=8079).

Communication channel	Total (n=8079), n (%)	Men, n (%; 95% CI^a^)	Age (years), mean (SD)	Low or middle education, n (%; 95% CI^a^)
Workplace	1641 (20.31)	163 (9.93; 8.58-11.48)	51 (9)	384 (23.82; 21.81-25.96)
Social media and internet	1819 (22.51)	399 (22; 20.09-23.89)	50 (11)	704 (39.55; 37.30-41.84)
Newspaper	1595 (19.74)	363 (22.76; 20.77-24.88)	55 (11)	578 (36.84; 34.49-39.25)
Relative or friend	1353 (16.74)	465 (34.37; 31.88-36.94)	55 (11)	602 (44.89; 42.25-47.56)
Health care	1069 (13.23)	260 (24.32; 21.84-26.98)	54 (11)	485 (45.84; 42.86-48.85)
StopDia	544 (6.73)	147 (27; 23.5-31.0)	56 (10)	205 (38.7; 34.6-42.9)
Radio or television	487 (6.03)	128 (26.3; 22.6-30.4)	58 (9)	221 (45.8; 41.4-50.2)
Pharmacy	287 (3.55)	61 (21.7; 17.3-26.9)	54 (11)	105 (36.7; 31.3-42.4)
NGO^b^	222 (2.74)	56 (25.2; 20.0-31.3)	53 (13)	89 (40.3; 34.0-46.9)
Event	149 (1.84)	27 (18.1; 12.8-25.1)	58 (10)	49 (33.3; 26.2-41.3)
Other	121 (1.49)	23 (19; 13.0-26.9)	53 (8)	29 (27.6; 20.0-36.9)
Multiple channels^c^	1084 (13.41)	248 (22.89; 20.51-25.50)	53 (11)	400 (37.31; 34.47-40.25)
All	8079 (100.00)	1815 (22.47; 21.58-23.40)	53 (11)	3003 (37.85; 36.79-38.92)

^a^Binomial variable CIs were calculated using the Wilson method.

^b^NGO: nongovernmental organization.

^c^Respondents could select multiple communication channels and were included in all mentioned categories.

### Effect of Active Versus Passive Recruitment

Of all respondents, 15.07% (5035/33,399) replied that they had been actively asked or recommended by somebody to determine their T2D risk. Active recruitment increased the likelihood of eligible respondents expressing interest in participating in StopDia, compared with passive recruitment (1808/4431, 40.8% vs 6268/19,814, 31.63%; *P*<.001). Active recruitment increased the likelihood among men (539/1638, 32.91% vs 1276/5609, 22.75%; *P*<.001) and among women (1269/2972, 45.45% vs 4992/14,204, 35.15%; *P*<.001) and across educational levels (low: 164/1788, 9.17% vs 536/6143, 8.72%; *P*=.01; middle: 608/1788, 34% vs 1695/6143, 27.59%; *P*<.001; high: 3912/6143, 63.68% vs 1016/1788, 56.82%; *P*<.001). Sex or educational level did not modify the differences between active and passive recruitment.

## Discussion

### Principal Findings

This study compared different communication channels with regard to their ability to reach people who are at risk of developing T2D and to engage them to take part in a T2D prevention study. A wide spectrum of channels was used, and some of them applied modern approaches, such as social media. The conversion rate (proportion of those who were eligible and willing to participate from the total number reached) of our recruitment (5882/33,399, 17.61%) was close to the rate achieved via recruitment through workplaces and media in previous eHealth studies [38].

Of those individuals who completed the web-based screening tool, 53.21% (13,925/26,167) were at risk, 30.01% (7877/26,167) were men, and 39.77% (10,136/25,485) had low or middle education. The largest absolute number of persons reached altogether and at risk was through social media and the internet, workplace, and newspapers. The proportion of at-risk people was the highest among those reached via community pharmacies (415/608, 68.3%) and health care (1631/2535, 64.33%).

A relative or friend was the communication channel that reached the largest percentage of men who were interested in participating in StopDia (1353/3979, 34%). Health care (578/1069, 54.07%) and radio or TV (225/487, 46.2%) reached the largest proportion of interested persons with lower education.

The PIPE framework provides steps that should be considered when designing and evaluating disease prevention programs [13]. Important indicators are *penetration* (the proportion of target group reached) and *participation* (the proportion of invited people who participated in the intervention). Of the residents aged 18-70 years in the study areas of North Savo (population 165,325), South Karelia (population 86,541) and Päijät-Häme (population 133,575) [39], a total of 33,399 people used our StopDia digital screening tool during the study period. This accounted for 8.67% (33,399/385,411) of the target population in these areas. On the basis of findings from a population-based survey [40], we can estimate that 1 in 4 adults (approximately 96,360) in these areas are at an increased risk of T2D. Consequently, we can calculate that our screening strategy managed to reach up to 14.44% (13,925/96,360) of the population at risk in the study areas within one year. Furthermore, of those who completed the screening, 53.21% (13,925/26,167) were at increased risk, which is twice as many as estimated in the general population. This suggests that our targeted communication strategy was able to reach the population segment at risk.

On the basis of our results, the selection of the most appropriate communication channels clearly depends on the primary goal of the outreach strategy. If the aim is to increase awareness among the general population, channels that reach the largest number of people should be used. If the aim is to find people at risk and engage them in preventive interventions, more personalized approaches may be useful. The largest number of respondents was obtained via social media, workplaces, and newspapers. These channels were thus effective in increasing population knowledge on the ongoing StopDia lifestyle intervention RCT and T2D risk factors in general. However, the proportion of people at risk among those reached via these channels was much smaller than that reached through pharmacies and health care. Workplace campaigns conducted via email can be especially effective in engaging people to test their risk, but they are not likely to reach those most at risk, thus decreasing health disparities [14].

In the planning phase, we assumed that health care providers and pharmacies would be the most important recruitment channels by incorporating opportunistic screening into their everyday activities. Therefore, we established an active network with local health care operators, produced and printed many materials, and organized training sessions for service providers and information days in pharmacies. This phase of organizational engagement is considered a pivotal part of this process [38,41]. However, it proved to be a challenge to integrate screening activities into the everyday work of health care professionals, and thus, the total number of respondents reached via these channels was low. On the other hand, among the people who were reached via health care and pharmacies, the proportion of individuals who had an increased risk for T2D and who were more likely to be interested in participating was the highest. Therefore, health care and pharmacy services could be important collaborators in targeted screening, but they cannot replace other channels that can reach other important population groups, such as people who use health care services infrequently. Thus, our findings suggest that risk screening should be a joint effort between different sectors of society, and it cannot be covered by the health care sector only. In addition, increasing public awareness and risk screening should be included in the health care sector as a defined function, as it proved to be a challenge to implement it as part of the existing services. It is important to consider that it also requires new skills from health care professionals, such as communication and marketing.

Interestingly, relatives and friends proved to be an important communication channel, especially for men, although the primary channel where the relatives themselves had received information about the StopDia project was not known. For example, our social media campaign “Have you seen this man?” aiming to reach men at T2D risk, produced a temporal peak in our screening tool visits, with most of the respondents being men. The valuable role of relatives and friends should be acknowledged while choosing channels and formulating recruitment messages in future campaigns.

Another a priori assumption was that personal prompting from somebody, such as a relative or a health care professional, would increase the likelihood of being interested in participating, as reported in a previous study [42]. This assumption was also true in our study. However, activities that required the study personnel to be present, such as community events and fairs, had very low overall reach and yield.

Our results also suggest that being exposed to the recruitment message via several communication channels increases interest in participation, compared with, for example, single media. Similar results have been observed in other large-scale lifestyle intervention recruitment studies [43]. Amplifying the reach of the message using multiple opportunistic approaches, such as recruitment through social media and journalistic media coverage, has been reported to be promising in the existing literature [44]. In general, formulating inclusive recruitment messages is crucial, especially in reaching the hard-to-reach populations [45]. Using an informal, warm tone of voice and relatable characters in promotional videos could have contributed to the success of the digital marketing efforts of our study.

Digital communications via social media may offer new ways to reach people who are often underrepresented in health interventions, such as the less-educated population groups and men [42,46]. Diabetes prevention research conducted in Australia, on the other hand, reported meager success in social media marketing [43]. Our results suggest that low threshold information delivered via digital channels is an efficient way to engage people with lower socioeconomic status. Lack of email address (or unwillingness to reveal it) may still be an important barrier for participation in interventions, including a digital component, especially among non–office-going men. Up to 12.94% (1802/13,925) of respondents at risk were deemed ineligible to participate in the StopDia study, and of them, 3 out of 4 were deemed ineligible because of a lack of email address. Among them, 37.01% (667/1802) of men and 47% (847/1802) of people with lower education were overrepresented, and their mean FINDRISC score was higher than that of all respondents (15.3 vs 12.0). This is an important finding, as men and less-educated people are in danger of being sidetracked in the future when new digital tools replace the traditional screening and intervention models. Thus, people from low socioeconomic groups might need financial aid to participate in prevention activities.

It must also be taken into account that part of the population faces difficulties with digital services and the most vulnerable people can lack internet access altogether; for example, 19% of Finnish persons with only a basic level of education have never used the internet [47]. In member states of the European Union, 43% of the population is reported to have less than basic digital skills [48].

Health policies and interventions can have greater efficacy among those with higher education than those with lower education [14,49]. There is also accumulating evidence that so-called high-agency population interventions based on traditional media campaigns and leaflets are likely to reinforce socioeconomic inequalities in health [50]. On the other hand, there seems to be no difference in the effectiveness of prevention interventions between educational groups, as long as they are reached and are participating in the programs [18,51]. On the basis of the results of our recruitment strategy, we now have useful information on ways to reach these underserved population groups.

The FINDRISC questionnaire was originally developed for use by both health care personnel and by people themselves, as a quick and simple tool to assess one’s risk of developing T2D within 10 years of age. Our study showed that the FINDRISC questionnaire can be used as a web-based tool to screen and recruit participants in a T2D prevention study. Not surprisingly, the FINDRISC question that was most often omitted was waist circumference, probably because people tend not to own a measuring tape. We were anticipating this and tried to overcome the problem by printing and distributing copious measuring tapes with FINDRISC printed on the reverse side, for example, in local pharmacies and exercise facilities. In the future, the necessity of waist circumference measurement in a web-based screening tool should be weighed against its effect on the test completion rate.

The most important caveat in our recruitment strategy was that we were not able to contact the respondents but had to rely on them being proactive in making the appointment with the study nurse, either over the phone or using a web-based system, depending on the area. As StopDia was a clinical trial, we could not collect any contact information from the respondents before they had signed an informed consent face-to-face with the study nurse. Of the eligible respondents, 48.52% (5882/12,123) were willing to participate in the StopDia study; however, only 3271 people attended the RCT baseline visit, of whom 11.07% (362/3271) were found to have previously undiagnosed T2D and were thus excluded from the StopDia RCT [52]. The number of randomized participants in the StopDia RCT was 2909, which is less than half of the number eligible and willing based on the screening site data. Even though the proportion is in line with findings from other studies [53,54], in future programs, special attention should be given to making the path from risk identification to intervention recruitment as smooth and effortless as possible, to improve the uptake of interventions at this important *window of opportunity* [55].

The strengths and limitations of our study must be addressed. Our study complements the scarce knowledge on the effectiveness of traditional marketing and digital campaigns to recruit participants representing different population groups in a T2D prevention study. We were able to reach a large proportion of the target population, and the ample data on real-life screening processes provide a rich source of research. The findings from the stepwise screening process are readily usable in prevention implementation programs.

As we were using a web-based screening tool with self-reported data, we saw a relatively large number of entries that were not completed, 21.66% (7232/33,399) of all respondents or where the entered data were not plausible. In addition, multiple responses from the same IP address were allowed, as the same device might be used by several people, for example, members of the same family or users of public service desks. It is thus likely that we may have had multiple answers from the same respondents included in the data. These limitations need to be considered when interpreting the results.

Importantly, we had no objective data on the communication channel and had to rely on respondents’ answers. The free-text answers to the communication channel question were not always clear, and the categorization into one of our selected categories was sometimes arbitrary. However, free-text answers were provided by only 4.84% (1616/33,399) of all persons who named a communication channel.

### Conclusions

We investigated the effectiveness of a large-scale traditional and digital marketing campaign to recruit participants in a T2D prevention study. With a comprehensive communication strategy that used several recruitment channels, we were able to reach a significant proportion of people with increased T2D risk in the study areas. Channels, such as social media and newspapers, that reach many people proved to be the most effective in risk identification. On the other hand, more personalized approaches increased the engagement of usually underrepresented groups, such as men and less-educated people. Health care services and pharmacies have reached people with a particularly high T2D risk. To increase recruitment and study enrollment, the screening path should be as smooth and effortless as possible for the user, avoiding transition points that will lead to the loss of eligible participants. To ensure the large-scale implementation of risk identification followed by preventive interventions, it is important to apply multiple different tactics to reach the target population as part of the existing service system.

## Appendix

Multimedia Appendix 1 StopDia recruitment campaign communications outputs and reach.

## Abbreviations

FINDRISC: Finnish Diabetes Risk Score
NGO: nongovernmental organization
PIPE: Penetration, Implementation, Participation, and Effectiveness
RCT: randomized controlled trial
T2D: type 2 diabetes
TV: television
