# Supplementary material for: Comparison of Communication Channels for Large-Scale Type 2 Diabetes Risk Screening and Intervention Recruitment: Empirical Study
Source: JMIR Diabetes. 2021 Sep 9;6(3):e21356. doi: 10.2196/21356 (PMC8461532; doi:10.2196/21356)
Supplement: Multimedia Appendix 1 [file diabetes_v6i3e21356_app1.pdf]

StopDia Recruitment Campaign Communications Outputs & Reach

Class	Direct costs	Content	Geographical targeting	Reach	Engagement
Television infospot	No	Infospot at the national TV	Finland	Potential 350 000	N/A
Television infospot	No	Infospot at the national TV	Finland	Potential 350 000	N/A
Total news, print and online	No	Interviews and news based on press releases total 5 (5 press releases)	Campaign area	N/A	N/A
Total news, television and radio	No	Interviews and news based on press releases (2 local television interviews, 4 local radios interviews)	Campaign area	N/A	N/A
Advertisement	Yes	Bus campaign	Päijät-Häme		N/A
Event	Yes	Hosting the icehockey game	North Savo	N/A	N/A
Advertisement, news	Yes	Print newpaper ad: South-Karelia, in total two ads, in two regional newspapers	South Karelia	Potential 84 000	N/A
Advertisement, news	Yes	Online newspaper ad: Kaakon Viesti	South Karelia	Potential 10 000	150
Total news, print and online	No	Interviews and news based on 5 press releases, total 122	Campaign area & Finland	Potential 400 000	N/A
Total news, television and radio	No	Interviews and news based on press releases (2 local television, 4 local radio)	Campaign area	N/A	N/A
Public sector cooperation	No	Employees of the public sector in are area	North Savo	16 000	N/A
NGO and Healthcare cooperation	No	NGO and Healthcare campaing start	Campaign area	N/A	N/A
E-mail pharmacies	No	Pharmacy campaign start	Campaign area	N/A	N/A
Newsletter to (600 people subscribed)	No	Recruitment starts	Campaign area	N/A	N/A
SMS	Yes	SMS to people in RCT, ask your friend	Campaign area	3000	N/A
Healthcare cooperation	No	Campaign to distribute flyer in healthcare	Campaign area	N/A	N/A
Total Youtube: Video	Yes	Video, two separate videos	Campaign area	673 439	160 714
Total Google ads banner	Yes	Ad view, 5 different ads	Campaign area	504 333	289
Print materials total	Yes	Flyers and poster, volume 150 000	Campaign area	N/A, volume 150 000	N/A
Facebook ads total	Yes	Video, ad with a picture, promoted post, total adset 50	Campaign area	617 925	18 574
Facebook organic and viral	No	Posts by the Page regional groups, sharing to collagues from personal accounts	Campaign area	N/A	N/A
Private sector cooperation	No	Print materal distribution 80 companies contacted	Campaign area	N/A	N/A

A summary of the main outputs during the campaing. The reach and engagement are not comparable, as different media has different metrics.
The recruitment took place in three areas: Päijät-Häme, South Karelia and North Savo. The outputs were targeted by default to all of them, labeled as Campaign area.
Activities were targeted geographically to only one area, if seen necessary. Some of the activities were not targeted, as the national news.
